# Waiting Time from Diagnosis to Treatment has no Impact on Survival in Patients with Esophageal Cancer

**DOI:** 10.1245/s10434-016-5191-6

**Published:** 2016-03-24

**Authors:** E. Visser, A. G. Leeftink, P. S. N. van Rossum, S. Siesling, R. van Hillegersberg, J. P. Ruurda

**Affiliations:** Department of Surgery, University Medical Center Utrecht, GA Utrecht, The Netherlands; Center for Healthcare Operations Improvement and Research, University of Twente, Enschede, The Netherlands; UMC Utrecht Cancer Center, University Medical Center Utrecht, Utrecht, The Netherlands; Department of Radiotherapy, University Medical Center Utrecht, Utrecht, The Netherlands; Department of Research, Netherlands Comprehensive Cancer Organization (IKNL), Amsterdam, The Netherlands; Department of Health Technology and Services Research, University of Twente, Enschede, The Netherlands

## Abstract

**Background:**

Waiting time from diagnosis to treatment has emerged as an important quality indicator in cancer care. This study was designed to determine the impact of waiting time on long-term outcome of patients with esophageal cancer who are treated with neoadjuvant therapy followed by surgery or primary surgery.

**Methods:**

Patients who underwent esophagectomy for esophageal cancer at the University Medical Center Utrecht between 2003 and 2014 were included. Patients treated with neoadjuvant therapy followed by surgery and treated with primary surgery were separately analyzed. The influence of waiting time on survival was analyzed using Cox proportional hazard analyses. Kaplan–Meier curves for short (<8 weeks) and long (≥8 weeks) waiting times were constructed.

**Results:**

A total of 351 patients were included; 214 received neoadjuvant treatment, and 137 underwent primary surgery. In the neoadjuvant group, the waiting time had no impact on disease-free survival (DFS) [hazard ratio (HR) 0.96, 95 % confidence interval (CI) 0.88–1.04; *p* = 0.312] or overall survival (OS) (HR 0.96, 95 % CI 0.88–1.05; *p* = 0.372). Accordingly, no differences were found between neoadjuvantly treated patients with waiting times of <8 and ≥8 weeks in terms of DFS (*p* = 0.506) and OS (*p* = 0.693). In the primary surgery group, the waiting time had no impact on DFS (HR 1.03, 95 % CI 0.95–1.12; p = 0.443) or OS (HR 1.06, 95 % CI 0.99–1.13; *p* = 0.108). Waiting times of <8 weeks versus ≥8 weeks did not result in differences regarding DFS (*p* = 0.884) or OS (*p* = 0.374).

**Conclusions:**

In esophageal cancer patients treated with curative intent by either neoadjuvant therapy followed by surgery or primary surgery, waiting time from diagnosis to treatment has no impact on long-term outcome.

Esophageal cancer is the eighth most common cancer and the sixth most common cause of cancer-related mortality worldwide.^1^,^2^ Surgical resection is the cornerstone of curative treatment with a 5-year survival rate of 34–36 % in patients treated with primary surgery.^2^ Multimodality treatment with neoadjuvant chemoradiotherapy or perioperative chemotherapy is increasingly applied and results in improved 5-year survival rates of 36–47 %.^3^–^6^

Waiting times have been shown to be an important quality indicator for cancer care.^7^ In the Netherlands, the period between diagnosis and treatment is currently recommended to be reduced to a maximum of 5 weeks, and all Dutch hospitals have been obliged to publish their waiting times once per month and submit these to the Dutch Healthcare authority.^8^,^9^ Waiting time before treatment is distressing for patients and impairs their quality of life.^10^–^12^ Several studies investigated the length of waiting time in esophageal cancer.^13^–^18^ However, these studies lacked survival analyses, are currently outdated, or only included patients treated with primary surgery.^13^–^18^ Therefore, literature about the impact of waiting time on the long-term outcome in patients with esophageal cancer is scarce.

This study was designed to determine the impact of waiting time from diagnosis to treatment on disease-free survival (DFS) and overall survival (OS) in patients with esophageal cancer undergoing esophagectomy with curative intent, either with or without neoadjuvant therapy.

## Materials and Methods

### Study Population

Data regarding 426 consecutive patients who were planned for esophagectomy with curative intent for esophageal cancer at the University Medical Center Utrecht (UMC Utrecht), from October 2003 to December 2014, were extracted from a prospectively collected database. Exclusion criteria consisted of emergency esophagectomies, participation in the Barrett’s esophagus surveillance program, detected unresectable tumor (cT4b) or metastatic disease (M1) intraoperatively, and in-hospital or 90-day postoperative mortality. Institutional review board approval was obtained, and the informed consent requirement was waived for this study.

### Pretreatment Workup

A substantial proportion of the patients were referred to the UMC Utrecht for treatment after their esophageal cancer diagnosis was established in a referring hospital. The remaining patients had the UMC Utrecht as their first-line hospital and were directly referred by a general practitioner (GP). In all patients, upper gastrointestinal endoscopy with biopsy was performed to confirm the diagnosis of esophageal cancer. Further investigations included endoscopic ultrasound (EUS), computed tomography (CT) scanning of thorax and abdomen, and ultrasonography of the neck. F-fluorodeoxyglucose positron emission tomography (FDG-PET) was not regularly used but has become part of the standard staging in our center since July 2013. All patients were discussed at a multidisciplinary tumor board in which a definitive treatment plan was constructed.

### Treatment

According to the Dutch guidelines, patients with locally advanced disease (cT ≥ 2 or cN+) received either perioperative chemotherapy or neoadjuvant chemoradiotherapy.^3^,^4^ Before June 2012, the standard treatment for patients with esophageal cancer in our center consisted of perioperative chemotherapy.^3^,^6^ From June 2012, patients received neoadjuvant chemoradiotherapy.^4^ Before 2008, neoadjuvant therapy was not part of the standard protocol and most patients were operated on without neoadjuvant therapy. In addition, primary surgical esophageal resection was performed in patients who were not eligible for neoadjuvant treatment. After esophagectomy with en bloc lymphadenectomy, all patients underwent gastric tube reconstruction with a cervical anastomosis. All patients were divided into one of two groups, including a group of patients who received neoadjuvant treatment followed by surgery, and a group of patients who underwent primary surgery.

### Follow-Up

Clinical and histopathological characteristics were retrieved from the database. The resected specimens were reviewed by pathologists in accordance with the 7th edition of the American Joint Committee on Cancer TNM staging system.^19^ A radical resection (R0) was defined using the College of American Pathologist criteria.^20^ Following hospital discharge, patients were followed at the outpatient clinic for 5 years. In case of clinical suspicion of tumor recurrence, diagnostic imaging was performed. For patients who were discharged after 5 years of follow-up, the general practitioner was contacted for additional information on recurrent disease. DFS was defined as the number of months from the start date of neoadjuvant therapy until the date of recurrent disease in the neoadjuvant group and from the date of surgery until the date of recurrent disease in the primary surgery group. OS was defined as the number of months from the start date of neoadjuvant therapy until the date of death or last follow-up in the neoadjuvant group and from the date of surgery until the date of death or last follow-up in the primary surgery group.

### Statistical Analysis

The distribution of continuous characteristics was reported as median [interquartile range (IQR)] or mean ± standard deviation (SD), and categorical variables were reported as numbers and percentages. For the neoadjuvantly treated group, waiting time was defined as the number of weeks from the date of diagnosis to the start date of neoadjuvant therapy. For the primary surgery group, waiting time was defined as the number of weeks from the date of diagnosis to the date of surgery. The date of the first upper gastrointestinal endoscopy, on which the diagnosis of esophageal cancer had been established by histology from biopsies, was used as date of diagnosis.

Spearman’s rank correlation coefficients (*ρ*) were calculated to determine whether waiting time from diagnosis to treatment was influenced by (i.e., correlated with) the baseline characteristics. Spearman’s ρ was interpreted as follows: a positive or negative correlation coefficient of 0.80–1.00 was considered very strong; 0.60–0.79, strong; 0.40–0.59, moderate; 0.20–0.39, weak; and 0–0.20, very weak.

The prognostic influence of the studied parameters, including waiting time and all baseline characteristics of DFS and OS, was assessed using univariable Cox proportional hazard analysis, which provided hazard ratios (HRs) along with 95 % confidence intervals (CIs). Studied parameters that yielded a *p* value <0.10 in univariable analysis were entered in multivariable Cox proportional hazards models, separately for DFS and OS. The original scale of waiting time was used for Cox regression analysis, because logarithmic transformation did not improve the proportional hazard assumption.

In accordance with literature and based on guidelines, waiting time was initially categorized into three groups: <5, 5–8, and ≥8 weeks.^9^,^18^ However, due to a limited number of patients, the number of groups was reduced to two groups with representative sample sizes, consisting of short and long time waiters (<8 and ≥8 weeks), respectively. Both groups were compared using the log-rank test after construction of Kaplan–Meier survival curves. All statistical analyses were performed using IBM SPSS Statistics Version 21 for Windows (IBM Corp., Armonk, NY). A *p* value <0.05 was considered statistically significant.

## Results

### Baseline Characteristics

Of the 426 patients who were planned for esophagectomy in the study period, 59 were excluded because of emergency esophagectomy (*n* = 3), participation in the Barrett’s esophagus surveillance program (*n* = 7), detected unresectable tumor (cT4b) or metastatic disease (M1) intraoperatively (*n* = 21), and in-hospital or 90-day postoperative mortality (*n* = 29). In 15 patients, waiting time could not be calculated, because the date of diagnosis or the starting date of neoadjuvant therapy was unknown. The remaining 351 patients were included in this study (Table 1).

**Table 1:** Baseline characteristics of 351 patients treated with neoadjuvant therapy followed by esophagectomy or primary surgery for esophageal cancer

	Neoadjuvant therapy total (*n* = 214) *n* (%)*	Primary surgery total (*n* = 137) *n* (%)*
Age at diagnosis, year [mean ± SD]	63 (8.4)	64 (9.9)
Gender		
Male	171 (80)	93 (68)
Female	43 (20)	44 (32)
ASA score		
1	49 (23)	35 (26)
2	136 (64)	70 (51)
≥3	29 (14)	32 (23)
Tumor location		
Upper esophagus	3 (1)	0 (0)
Middle esophagus	30 (14)	29 (21)
Distal esophagus	105 (49)	62 (45)
Gastroesophageal junction	76 (36)	46 (34)
Neoadjuvant therapy		
None	0 (0)	137 (100)
Chemotherapy	103 (48)	0 (0)
Chemoradiotherapy	111 (52)	0 (0)
Surgical approach		
Transthoracic	176 (82)	84 (61)
Transhiatal	38 (18)	53 (39)
Histological type		
Adenocarcinoma	175 (82)	92 (67)
Squamous cell carcinoma	39 (18)	44 (32)
Other	0 (0)	1 (1)
pT stage		
T0	39 (18)	2 (2)
T1-2	64 (30)	39 (28)
T3-4a	111 (51)	96 (70)
pN stage		
N0	108 (51)	46 (34)
N1	54 (25)	39 (29)
N2-3	52 (24)	52 (38)
Radicality		
R0	198 (93)	117 (85)
R1	16 (8)	20 (15)
Referral		
By general practitioner	23 (11)	34 (25)
By another hospital	191 (89)	103 (75)
Year of diagnosis		
2003–2005	2 (1)	34 (25)
2006–2008	22 (10)	50 (36)
2009–2011	91 (43)	33 (24)
2012–2014	99 (46)	20 (15)

*SD* standard deviation, *ASA* American Society of Anesthesiologists

* Percentages may not add up to 100 due to rounding

The number of patients in the neoadjuvant group was 214. Mean age at diagnosis was 63 years ± 8.4 (SD). The majority of patients was male (*n* = 171, 80 %), received neoadjuvant chemoradiotherapy (*n* = 111, 52 %), underwent transthoracic esophagectomy (*n* = 176, 82 %), and was pathologically staged as ypT3-4a (*n* = 111, 51 %), ypN0 (*n* = 108, 51 %), and R0 (*n* = 198, 93 %). The primary surgery group consisted of 137 patients. Mean age at diagnosis was 64 years ± 9.9 (SD). The majority of patients was male (*n* = 93, 68 %), underwent transthoracic esophagectomy (*n* = 84, 61 %), and was pathologically staged as pT3-4a (*n* = 96, 70 %), pN+ (*n* = 91, 66 %), and R0 (*n* = 117, 85 %).

### Waiting Time and Baseline Characteristics

The median time from diagnosis to neoadjuvant treatment was 6.4 weeks (IQR 5.3–7.7) for patients in the neoadjuvant group. Spearman’s rank correlation coefficients showed weak to very weak correlations of waiting time with all baseline characteristics ranging from −0.22 to +0.16 (Table 2). In the primary surgery group, patients had a median time from diagnosis to surgery of 9.7 weeks (IQR 8.0–13.1). Spearman’s rank correlation coefficients showed weak to very weak correlations of waiting time with baseline characteristics ranging from −0.22 to +0.25 (Table 2). The median waiting time of excluded patients detected with an unresectable tumor (cT4b) or metastatic disease (M1) intraoperatively (*n* = 21) was 6.1 weeks (IQR 4.7–11.0) in the neoadjuvant group (*n* = 7) and 8.8 weeks (IQR 4.2–12.4) in the primary surgery group (*n* = 14).

**Table 2:** Spearman’s rank correlation coefficients (*ρ*) for waiting time for patients treated with neoadjuvant therapy followed by esophagectomy or primary surgery for esophageal cancer

	Neoadjuvant therapy	Primary surgery
Age at diagnosis	0.11	0.25
Gender	0.02	0.03
ASA score	0.16	0.14
Tumor location	0.05	−0.03
Type of neoadjuvant therapy	−0.08	n.a.
Surgical approach	0.02	0.22
Histological type	0.01	0.01
pT stage	0.06	−0.22
pN stage	0.07	−0.11
Radicality	0.01	0.09
Referral	0.16	0.10
Year of diagnosis	−0.22	0.17

*ASA* American Society of Anesthesiologists, *n.a.* not applicable

Positive or negative coefficients indicate positive or negative correlations with 1 or −1 being the strongest positive or negative relationship

### Waiting Time and Survival

For surviving patients in the neoadjuvant group, the median follow-up was 28 months (range 7–75). Of the 214 patients, 102 (48 %) developed recurrent disease during follow-up. Median DFS was 16 months (range 3–75), whereas median OS was 19 months (range 5–77). The OS rates at 1 and 3 years were 84 and 48 %, respectively. Univariable analyses of all studied variables in relation to DFS and OS are presented in Appendix Table 5. Waiting time did not influence significantly the DFS (HR 0.96, 95 % CI 0.88–1.04; *p* = 0.312) or OS (HR 0.96, 95 % CI 0.88–1.05; *p* = 0.372; Table 3). For DFS, multivariable analysis identified ypT3-4a stage, ypN+ stage, and irradicality as independent and significant prognostic factors associated with worse DFS. For OS, ypN+ stage remained independently and significantly associated with worse OS in multivariable analysis. In addition, a stratified analysis for type of neoadjuvant therapy showed that waiting time did not influence OS significantly in patients treated with chemotherapy (HR 0.92, 95 % CI 0.81–1.03; *p* = 0.159) or in patients treated with chemoradiotherapy (HR 1.05, 95 % CI 0.90–1.23; *p* = 0.509).

**Table 3:** Univariable and multivariable analysis of influence of waiting time on disease-free survival and overall survival in patients treated with neoadjuvant therapy followed by esophagectomy for esophageal cancer

	Univariable analysis			Multivariable analysis		
	HR	95 % CI	*p* value	HR	95 % CI	*p* value
Disease-free survival						
Additional week waiting time	0.96	0.88–1.05	0.362	0.96	0.88–1.04	0.312
ypT stage						
T0	Reference	–	–	Reference		
T1-2	1.55	0.69–3.47	0.288	1.22	0.54–2.79	0.635
T3-4a	3.94	1.89–8.25	**<0.001**	2.40	1.10–5.22	**0.028**
ypN stage						
N0	Reference	–	–	Reference	–	–
N1	1.83	1.11–3.03	**0.019**	1.72	1.03–2.88	**0.039**
N2-3	4.42	2.77–7.03	**<0.001**	3.09	1.81–5.25	**<0.001**
Radicality						
R0	Reference	–	–	Reference	–	–
R1	4.41	2.46–7.91	**<0.001**	1.93	1.00–3.70	**0.049**
Overall survival						
Additional week waiting time	1.00	0.91–1.08	0.910	0.96	0.88–1.05	0.372
ypT stage						
T0	Reference	–	–	Reference	–	–
T1-2	0.95	0.45–2.02	0.895	0.71	0.33–1.54	0.385
T3-4a	2.54	1.31–4.94	**0.006**	1.46	0.71–3.00	0.300
ypN stage						
N0	Reference	–	–	Reference	–	–
N1	2.07	1.25–3.43	**0.005**	2.04	1.21–3.43	**0.007**
N2-3	4.15	2.57–6.69	**<0.001**	3.04	1.74–5.33	**<0.001**
Radicality						
R0	Reference	–	–	Reference	–	–
R1	4.40	2.46–7.87	**<0.001**	1.90	0.99–3.66	0.055

*HR* hazard ratio, *CI* confidence interval

Analysis was performed using Cox regression model. Bold values indicate statistically significant results (e.g., *p* < 0.05). Only waiting time and variables with *p* < 0.1 from univariable analysis are reported and were used for multivariable analysis

For surviving patients in the primary surgery group, the median follow-up was 56 months (range 10–135). During follow-up, 67 of 137 patients (49 %) developed recurrent disease. Median DFS in the primary surgery group was 18 months (range 2–133), whereas median OS was 23 months (range 2–135). The OS rates at 1, 3, and 5 years were 80, 50, and 45 %, respectively. Univariable analyses of all studied variables in relation to DFS and OS are presented in Appendix Table 6. Waiting time from diagnosis to surgery did not influence significantly DFS (HR 1.03, 95 % CI 0.95–1.12; *p* = 0.443) or OS (HR 1.06, 95 % CI 0.99–1.13; *p* = 0.108; Table 4). For DFS, multivariable analysis identified pT3-4a stage and pN+ stage as independent and significant prognostic factors associated with worse DFS. For OS, pN+ stage and irradicality remained independently and significantly associated with worse OS in multivariable analysis.

**Table 4:** Univariable and multivariable analysis of influence of waiting time on disease-free survival and overall survival in patients treated with primary surgery for esophageal cancer

	Univariable analysis			Multivariable analysis		
	HR	95 % CI	*p* value	HR	95 % CI	*p* value
Disease-free survival						
Additional week waiting time	0.97	0.91	0.222	1.03	0.95	0.443
ASA score						
1	Reference	–	–	Reference	–	–
2	0.63	0.37–1.09	0.100	0.95	0.53–1.72	0.876
≥3	0.54	0.27–1.07	0.075	0.95	0.32–1.39	0.284
Surgical approach						
Transthoracic	Reference	–	–	Reference	–	–
Transhiatal	0.50	0.29–0.86	**0.012**	0.66	0.36–1.22	0.182
pT stage						
T0-2	Reference	–	–	Reference	–	–
T3-4a	4.08	2.02–8.26	**<0.001**	2.51	1.13–5.57	**0.024**
pN stage						
N0	Reference	–	–	Reference	–	–
N1	2.89	1.35–6.20	**0.006**	2.25	1.00–5.05	**0.029**
N2-3	5.20	2.58–10.50	**<0.001**	2.92	1.33–6.45	**0.008**
Radicality						
R0	Reference	–	–	Reference	–	–
R1	2.95	1.67–5.21	**<0.001**	1081	0.97–3.37	0.063
Additional year date diagnosis	0.88	0.81–0.97	**0.009**	0.94	0.85–1.05	0.266
Overall survival						
Additional week waiting time	0.99	0.95–1.05	**0.803**	1.06	0.99–1.13	0.108
Tumor location						
Middle esophagus	Reference	–	–	Reference	–	–
Distal esophagus	0.68	0.37–1.24	0.211	0.54	0.28–1.03	0.060
Gastro-esophageal junction	1.74	0.96–3.15	0.067	1.12	0.60–2.09	0.728
pT stage						
T0-2	Reference	–	–	Reference	–	–
T3-4a	2.95	1.62–5.38	**<0.001**	1.86	0.92–3.78	0.086
pN stage						
N0	Reference	–	–	Reference	–	–
N1	2.89	1.52–5.49	**0.001**	2.76	1.40–5.45	**0.004**
N2-3	3.25	1.78–5.94	**<0.001**	2.37	1.16–4.84	**0.018**
Radicality						
R0	Reference	–	–	Reference	–	–
R1	2.65	1.56–4.51	**<0.001**	2.08	1.17–3.70	**0.013**
Additional year date diagnosis	0.92	0.84–1.01	**0.070**	0.97	0.88–1.08	0.584

Analysis was performed using Cox regression model. Bold values indicate statistically significant results (e.g., *p* < 0.05). Only waiting time and variables with *p* < 0.1 from univariable analysis are reported and were used for multivariable analysis

*ASA* American Society of Anesthesiologists, *HR* hazard ratio, *CI* confidence interval

### Impact of Short versus Long Waiting Time on Long-term Survival

**Fig. 1:**
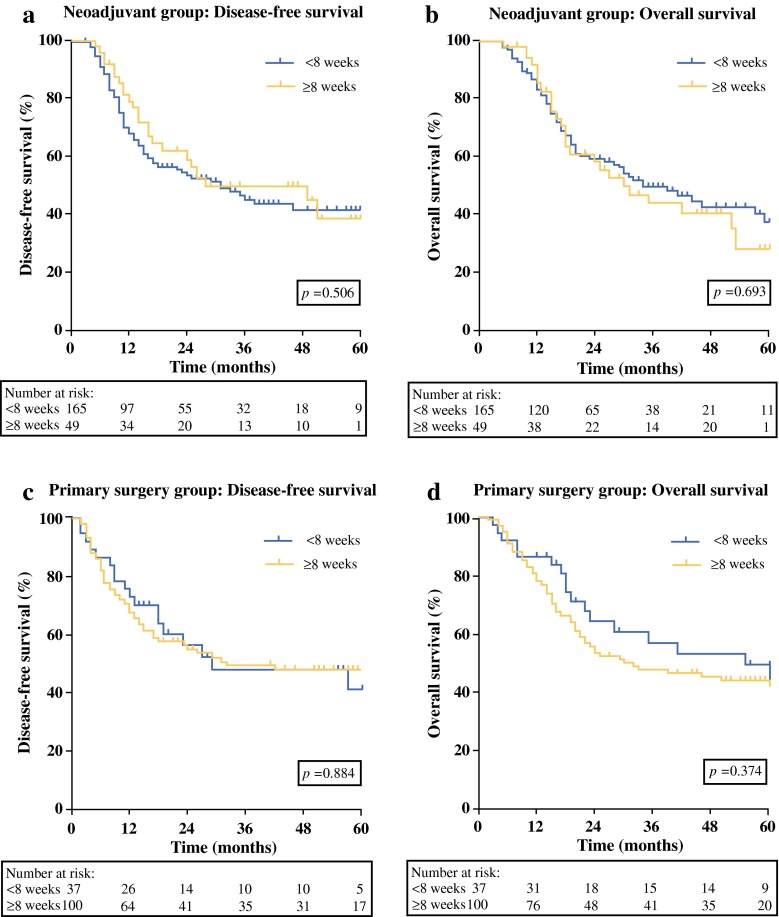
The influence of short (<8 weeks) and long (≥8 weeks) waiting time on disease-free survival (**a, c**) and overall survival (**b, d**) in patients treated with neoadjuvant therapy followed by surgery (**a, b**), and patients treated with primary surgery (**c, d**) for esophageal cancer. Survival curves were plotted by the Kaplan–Meier method

In the neoadjuvant group, no significant differences were found between patients with short (<8 weeks, *n* = 165) and long (≥8 weeks, *n* = 49) waiting times regarding DFS (*p* = 0.506) and OS (*p* = 0.693; Figs. 1a, b). In addition, in the primary surgery group, short (*n* = 37) and long (*n* = 100) waiting times did not result in significant differences in terms of DFS (*p* = 0.884) or OS (*p* = 0.374; Figs. 1c, d).

## Discussion

This single-center cohort study investigated the influence of waiting time on long-term outcome in patients who underwent esophagectomy with curative intent for esophageal cancer, either treated with neoadjuvant therapy or treated with primary surgery. This study showed that waiting time did not significantly impact long-term outcome for both patient groups.

This is the first study to demonstrate that waiting time does not significantly affect long-term outcome regarding DFS and OS for patients treated with neoadjuvant therapy followed by surgery for esophageal cancer. In line with previous studies, waiting time did not impact DFS or OS in patients treated with primary surgery.^17^,^18^ The majority of the included patients were treated within a time frame of approximately 3 months. Therefore, the findings of this study reflect the fact that if patients are treated within a clinically relevant waiting period, early treatment does not improve survival.

Because multimodality treatment was introduced recently as the standard of care, the median follow-up duration of 28 months is relatively short in the neoadjuvant group. However, median time to recurrence is 9 months and the majority of patients develop recurrent disease within 2 years after surgery.^21^,^22^ Therefore, the follow-up duration of the neoadjuvant group in this study was considered sufficient to assess accurately the impact of waiting time on both DFS and OS.

To provide more insight into the influence of waiting time on long-term outcome, waiting time was initially categorized into three groups in this study according to Grotenhuis et al.^18^ Due to a limited number of patients, two rather than three groups, consisting of patients with short (<8 weeks) and long (≥8 weeks) waiting times, were composed. This cutoff was used to create two groups with representative sample sizes. The results showed no difference in survival regarding DFS and OS for patients with waiting times of <8 weeks from diagnosis to the start of neoadjuvant therapy. In addition, for patients treated with primary surgery, no differences were found between short and long waiting times on long-term outcome.

In this study, waiting time was not influenced by one or more baseline characteristics. All correlations were weak to very weak (Spearman’s rank correlation coefficient <0.4). This finding suggests that a longer waiting time did not result in worse tumor characteristics with more advanced disease or in a smaller rate of radical tumor resections. Patients with shorter and longer waiting times had comparable pTN stages and R0 resections. For patients treated with neoadjuvant therapy, the waiting time did not differ between the type of neoadjuvant therapy (neoadjuvant chemoradiotherapy or perioperative chemotherapy).

In other types of cancer, several studies reported mixed findings about the potential association between waiting time and long-term survival. For breast cancer, studies mainly reported better outcomes with shorter waiting time, whereas in lung cancer, mixed findings of positive, negative, and no associations were reported.^23^–^27^ For pancreatic and colorectal cancer, most studies reported no associations between waiting time and survival.^28^–^31^

Numerous reports showed that increased waiting time results in major psychosocial stress in cancer patients. Patients prefer to be treated as soon as possible for the fear of tumor progression.^32^–^34^ Until hard conclusions can be drawn on the effect of waiting time on patients’ prognosis, efforts should be made to keep waiting time to a minimum. In the current study, the median waiting time between diagnosis and neoadjuvant treatment was 6.4 weeks, whereas in patients treated with primary surgery it was 9.7 weeks. The waiting time reflects resource availability and efficiency, which indicates that there is still room for improvement.^35^ However, the main reason for the long waiting time should be sought, because we are a tertiary referral center. To reduce waiting time, a more efficient diagnostic workup for patients with suspected esophageal cancer was recently introduced in our hospital, including rapid diagnostic pathways and frequent multidisciplinary team meetings for the establishment of a definitive treatment plan for each individual patient.^36^ This diagnostic workup resulted in a reduced median waiting time of 6.1 weeks for the neoadjuvant group and 8.1 weeks for the primary surgery group during the period of 2012–2014 (data not shown). Further steps still need to be taken to reduce the waiting time from the determination of the treatment plan until the start of treatment. The waiting time of the primary surgery group in this study is likely caused by the complexity of esophageal surgery planning. Esophagectomies are highly complex procedures with a median surgery duration of 6 h for transthoracic resection and therefore put a lot of pressure on operating room schedules.^37^

In our center, patients do not undergo standard disease evaluation after initial diagnosis of esophageal cancer until esophagectomy, and therefore possible tumor progression could only be detected during surgery. The median waiting time of 21 excluded patients detected with an unresectable tumor (cT4b) or metastatic disease (M1) intraoperatively and was comparable with the included patients who underwent esophagectomy with curative intent. Therefore, no significant selection bias of these patients is expected.

Of note, this study contains information about in-hospital waiting time only. Because patient waiting time (i.e., time between onset of symptoms and presentation to the GP) and doctor waiting time (i.e., time between presentation to the GP and endoscopy) account for a larger part of the total waiting time, these periods may still have an influence on long-term outcome.^13^,^15^,^18^ It would be interesting to identify what time frame is appropriate and whether a delay of any length does negatively influence long-term oncologic outcomes. Unfortunately, this study was not designed for such analysis.

In conclusion, this is the first study to demonstrate that waiting time does not impact long-term outcome in patients treated with neoadjuvant therapy combined with surgical resection for curable esophageal cancer. Furthermore, a longer waiting time did not affect the oncologic outcome for patients treated with primary surgery.

## Appendix

See Tables 5 and 6.

**Table 5:** Univariable analysis of influence of waiting time on disease-free survival and overall survival in patients treated with neoadjuvant therapy followed by esophagectomy for esophageal cancer

	Disease-free survival			Overall survival		
	HR	95 % CI	*p* value	HR	95 % CI	*p* value
Univariable analysis						
Additional week waiting time	0.96	0.88–1.05	0.362	1.00	0.91–1.08	0.910
Age at diagnosis	1.01	0.91–1.13	0.805	1.01	0.98–1.03	0.503
Gender						
Female	Reference	–	–	Reference	–	–
Male	1.28	0.77–2.13	0.342	1.01	0.62–1.65	0.962
ASA classification						
1	Reference	–	–	Reference	–	–
2	0.84	0.52–1.33	0.452			0.389
>3	1.33	0.72–2.47	0.366	0.81	–	0.480
Tumor location						
Upper esophagus	Reference	–	–	Reference	–	–
Middle esophagus	0.59	0.12–3.06	0.534	0.61	0.13–2.83	0.524
Distal esophagus	0.87	0.19–3.95	0.853	0.74	0.18–3.05	0.677
Gastroesophageal junction	0.70	0.15–3.24	0.647	0.53	0.13–2.23	0.389
Neoadjuvant therapy						
Chemotherapy	Reference	–	–	Reference	–	–
Chemoradiotherapy	0.90	0.60–1.34	0.594	0.96	0.63–1.46	0.849
Surgical approach						
Transthoracic	Reference	–	–	Reference	–	–
Transhiatal	0.75	0.44–1.26	0.272	0.72	0.43–1.22	0.226
Histological type						
Adenocarcinoma	Reference	–	–	Reference	–	–
Squamous cell carcinoma	0.87	0.49–1.53	0.621	1.45	0.85–2.45	0.172
pT stage						
T0	Reference	–	–	Reference	–	–
T1-2	1.55	0.69–3.47	0.288	0.95	0.45–2.02	0.895
T3-4a	3.94	1.89–8.25	<0.001	2.54	1.31–4.94	0.006
pN stage						
N0	Reference	–	–	Reference	–	–
N1	1.83	1.11–3.03	0.019	2.07	1.25–3.43	0.005
N2-3	4.42	2.77–7.03	<0.001	4.15	2.57–6.69	<0.001
Radicality						
R0	Reference	–	–	Reference	–	–
R1	4.41	2.46–7.91	<0.001	4.40	2.46–7.87	<0.001
Referral						
By general practitioner	Reference	–	–	Reference	–	–
By another hospital	0.94	0.52–1.72	0.847	0.93	0.53–1.78	0.928
Additional year date diagnosis	1.01	0.91–1.13	0.805	1.07	0.95–1.21	0.250

Analysis was performed using Cox regression model

*ASA* American Society of Anesthesiologists, *HR* hazard ratio, *CI* confidence interval

**Table 6:** Univariable analysis of influence of waiting time on disease-free survival and overall survival in patients treated with esophagectomy for esophageal cancer

	Disease-free survival			Overall survival		
	HR	95 % CI	*p* value	HR	95 % CI	*p* value
Univariable analysis						
Additional week waiting time	0.97	0.91–1.02	0.222	0.99	0.95–1.05	0.803
Age at diagnosis	0.98	0.96–1.01	0.129	1.00	0.98–1.02	0.876
Gender						
Female	Reference	–	–	Reference	–	–
Male	1.27	0.75–2.16	0.379	1.02	0.63–1.66	0.932
ASA classification						
1	Reference	–	–	Reference	–	–
2	0.63	0.37–1.09	0.100	0.93	0.54–1.59	0.782
>3	0.54	0.27–1.07	0.075	0.98	0.53–1.83	0.984
Tumor location						
Middle esophagus	Reference	–	–	Reference	–	–
Distal esophagus	0.25	0.36–1.30	0.246	0.68	0.37–1.24	0.211
Gastro-esophageal junction	0.18	0.82–2.89	0.177	1.74	0.96–3.15	0.067
Surgical approach						
Transthoracic	Reference	–	–	Reference	–	–
Transhiatal	0.50	0.29–0.86	0.012	0.77	0.48–1.24	0.285
Histological type						
Adenocarcinoma	Reference	–	–	Reference	–	–
Squamous cell carcinoma	0.74	0.43–1.26	0.264	0.93	0.57–1.49	0.748
Other	3.33	0.45–24.45	0.238	3.37	0.46–24.77	0.233
pT stage						
T0-2	Reference	–	–	Reference	–	–
T3-4a	4.08	2.02–8.26	<0.001	2.95	1.62–5.38	<0.001
pN stage						
N0	Reference	–	–	Reference	–	–
N1	2.89	1.35–6.20	0.006	2.89	1.52–5.49	0.001
N2-3	5.20	2.58–10.50	<0.001	3.25	1.78–5.94	<0.001
Radicality						
R0	Reference	–	–	Reference	–	–
R1	2.95	1.67–5.21	<0.001	2.65	1.56–4.51	<0.001
Referral						
By general practitioner	Reference	–	–	Reference	–	–
By another hospital	1.50	0.82–2.75	0.190	1.00	0.60–1.67	0.999
Additional year date diagnosis	0.88	0.81–0.97	0.009	0.92	0.84–1.01	0.070

Analysis was performed using Cox regression model

*ASA* American Society of Anesthesiologists, *HR* hazard ratio, *CI* confidence interval
